# Transcriptomal dissection of soybean circadian rhythmicity in two geographically, phenotypically and genetically distinct cultivars

**DOI:** 10.1186/s12864-021-07869-8

**Published:** 2021-07-10

**Authors:** Yanlei Yue, Ze Jiang, Enoch Sapey, Tingting Wu, Shi Sun, Mengxue Cao, Tianfu Han, Tao Li, Hai Nian, Bingjun Jiang

**Affiliations:** 1 College of Life Sciences, Henan Agricultural University, 450002 Zhengzhou, China; 2 State Key Laboratory for Conservation and Utilization of Subtropical Agro-Bioresources, South China Agricultural University, 510642 Guangzhou, China; 3 MARA Key Lab of Soybean Biology (Beijing), Institute of Crop Sciences, The Chinese Academy of Agricultural Sciences, 100081 Beijing, China

**Keywords:** Soybean, Circadian rhythmicity, Time-series transcriptome, *J/GmELF3a*, *GmFT* family

## Abstract

**Background:**

In soybean, some circadian clock genes have been identified as loci for maturity traits. However, the effects of these genes on soybean circadian rhythmicity and their impacts on maturity are unclear.

**Results:**

We used two geographically, phenotypically and genetically distinct cultivars, conventional juvenile Zhonghuang 24 (with functional *J/GmELF3a*, a homolog of the circadian clock indispensable component *EARLY FLOWERING 3*) and long juvenile Huaxia 3 (with dysfunctional *j/Gmelf3a*) to dissect the soybean circadian clock with time-series transcriptomal RNA-Seq analysis of unifoliate leaves on a day scale. The results showed that several known circadian clock components, including *RVE1*, *GI*, *LUX* and *TOC1*, phase differently in soybean than in *Arabidopsis*, demonstrating that the soybean circadian clock is obviously different from the canonical model in *Arabidopsis*. In contrast to the observation that *ELF3* dysfunction results in clock arrhythmia in *Arabidopsis*, the circadian clock is conserved in soybean regardless of the functional status of *J*/*GmELF3a*. Soybean exhibits a circadian rhythmicity in both gene expression and alternative splicing. Genes can be grouped into six clusters, C1-C6, with different expression profiles. Many more genes are grouped into the night clusters (C4-C6) than in the day cluster (C2), showing that night is essential for gene expression and regulation. Moreover, soybean chromosomes are activated with a circadian rhythmicity, indicating that high-order chromosome structure might impact circadian rhythmicity. Interestingly, night time points were clustered in one group, while day time points were separated into two groups, morning and afternoon, demonstrating that morning and afternoon are representative of different environments for soybean growth and development. However, no genes were consistently differentially expressed over different time-points, indicating that it is necessary to perform a circadian rhythmicity analysis to more thoroughly dissect the function of a gene. Moreover, the analysis of the circadian rhythmicity of the *GmFT* family showed that *GmELF3a* might phase- and amplitude-modulate the *GmFT* family to regulate the juvenility and maturity traits of soybean.

**Conclusions:**

These results and the resultant RNA-seq data should be helpful in understanding the soybean circadian clock and elucidating the connection between the circadian clock and soybean maturity.

**Supplementary Information:**

The online version contains supplementary material available at 10.1186/s12864-021-07869-8.

## Background

The external environment changes with the day and night cycle. To adapt to such regular alterations, organisms including Bacteria, Archaea and Eukaryotes have developed various, elaborate inner time-keeping mechanisms, known as circadian clocks [1]. Circadian clocks perceive environmental time cues, with light being the most dominant one, and generate a 24-h diurnal rhythmicity by central oscillators to synchronize biological processes with daily changes [2–5].

In the model plant *Arabidopsis thaliana*, more than 20 clock related components have been identified, such as CIRCADIAN CLOCK ASSOCIATED 1 (CCA1), LATE ELONGATED HYPOCOTYL (LHY), PSEUDO RESPONSE REGULATOR 5 (PRR5), PRR7, PRR9, GIGANTEA (GI), TIMING OF CAB EXPRESSION 1 (TOC1/PRR1), LUX ARRHYTHMO (LUX), EARLY FLOWERING 3 (ELF3) and ELF4 [6, 7]. These components modulate each other at different time points to form morning-, afternoon-, and evening-phased interlocking transcriptional-translational feedback loops to make up a complex circadian clock network [6]. Moreover, ELF4, ELF3, and LUX can form a tripartite complex, the evening complex (EC), which features expression levels that peak at dusk. Significantly, the EC is essential in maintaining regular circadian rhythms, and its dysfunction results in clock arrhythmia [8–11].

ELF3, a highly conserved plant-specific nuclear protein, is an indispensable component of the circadian clock. First, it works as a scaffold to directly bind ELF4 and LUX to form the EC [12, 13]. ELF3 can regulate the components of the circadian clock directly or indirectly, however it is regulated by negative feedback. Moreover, ELF3 can interact with various proteins that have distinct roles, including all types of phytochromes (PHYA-PHYE), E3 ubiquitin ligase CONSTITUTIVELY PHOTOMORPHOGENIC 1 (COP1), b-box transcription factor BBX19, bHLH transcription factor PIF4, MADS-box transcription factor SHORT VEGETATIVE PERIOD (SVP), LUX homologous protein NOX, MUT9-like nuclear kinases MLK1-4, circadian clock morning protein TOC1 and photoperiod pathway key protein GI [6]. Thus, ELF3 functions as a key hub, linking circadian clocks with other biological processes to orchestrate growth and development with the external environment.

ELF3 has essential functions in crops. Its monocot homologs *BdELF3* and *SvELF3* can functionally complement loss of *ELF3* in the dicot *Arabidopsis* [14]. Both *Earliness per se-D1* (*Eps-D1*) in bread wheat (*Triticum aestivum*) and *Eps-A*^*m*^*1* in *T. monococcum* [15] were proposed to be *ELF3* genes. *OsELF3.1* promotes flowering through inhibiting the phytochrome signaling pathway [16, 17] and leaf senescence in rice but delays leaf senescence in *Arabidopsis* [19].

Soybean is a short-day photoperiod-sensitive crop. Maturity is the most important trait in soybean breeding and production. Twelve maturity loci – *E1*-*E11* and *J* – have been proposed, and some have been identified [20]. Recently, Fu et al. [21] proposed some yet-unmapped maturity QTLs related to the south-to-north extension of Northeast China soybean. Notably, most known maturity loci are homologs of circadian clock genes. *E3* [22] and *E4* [23] encode phytochrome PHYA, which is involved in the input pathway of the circadian clock. *E2* [24] and *J* [25, 26] are respectively homologous to the afternoon-phased oscillator *GI* and evening-phased *ELF3*. A PRR family member GmPRR37/GmPRR3b was also recently identified as related to photoperiodic flowering and regional adaptation [27–29]. Wang et al. found that loss-of-function of clock gene *GmLCL* homologs leads to a late-flowering phenotype [4]. Therefore, the circadian clock should be involved in the regulation of maturity. However, the nature of the circadian clock in soybean is still unclear.

The *GmFT* (*FLOWERING LOCUS T*) family has essential functions in flowering and maturity. It has at least ten members in the soybean genome due to three whole-genome duplications proposed to occur over evolutionary history [30, 31]. Some members such as *GmFT2a*, *GmFT2b* and *GmFT5a* exert a conserved role as with *FT* in *Arabidopsis* and *Hd3a* in rice to promote flowering [30, 32–34]. Other members such as *GmFT1a* and *GmFT4* are neofunctionalized to inhibit flowering [35, 36]. Consistently, genetic analysis showed that *GmFT2a* and *GmFT4* are the maturity alleles *E9* and *E10*, respectively [37, 38]. CRISPR/Cas9 studies further provided knockout evidence to demonstrate the essential role of the *GmFT* family in flowering and maturity [39–41]. A seesaw model was proposed, where flowering promoter members *GmFT2a*/*GmFT5a* and flowering inhibitor members *GmFT1a*/*GmFT4* function antagonistically to determine the direction of soybean development [36]. However, although these genes are regulated by different photoperiod conditions, how the circadian clock regulates *GmFT* family is yet unclear.

In this study, we used RNA-Seq time-series transcriptomal data to analyze the circadian clock of soybean. Moreover, to better avoid potential bias resulting from a specific genetic background, we used two geographically and phenotypically distinct soybean cultivars, the conventional-juvenile cultivar ‘Zhonghuang 24’ (ZH24) from North China and the long-juvenile cultivar ‘Huaxia 3’ (HX3) from South China. Most importantly, these cultivars are genetically distinct because they differ by > 1.6 million genetic variations at the whole genome level [26]. Of these, one single-nucleotide deletion causes a loss-of-function mutation in *J/GmELF3a* and confers long juvenility to HX3. First, the soybean circadian clock was dissected at the transcriptomal level using RNA-Seq analysis then verified at the level of several individual circadian clock-related genes by qPCR analysis. Second, the oscillation rhythmicity was explored at the levels of gene expression, alternative splicing and chromosome activation. Third, differentially expressed genes (DEGs) were screened at different time points. Finally, the rhythmicity of the *GmFT* family was further analyzed and one model of *GmELF3a* regulating the *GmFT* family was proposed. This time-series transcriptome analysis provides a new perspective for the study of plant circadian rhythms, and suggests that rhythmicity is widespread at different levels. The findings are valuable for exploring the gene expression rhythmicity of soybean.

## Results

### Soybean exhibits a different circadian clock from the known canonical *Arabidopsis* model

**Fig. 1:**
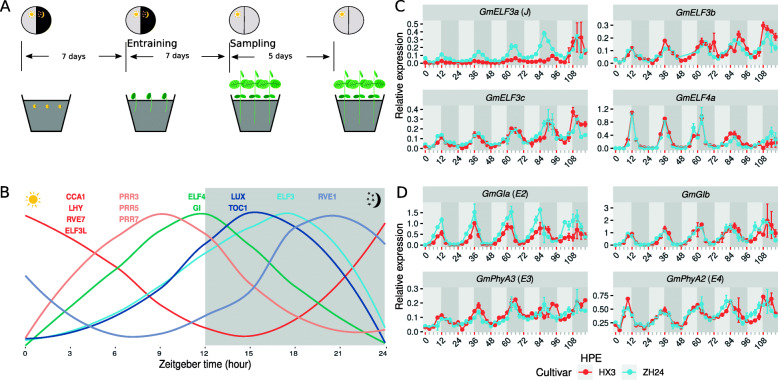
The soybean circadian clock is stable regardless of *GmELF3a* functionality. (**A**) Schematic illustration for the experimental design to entrain soybean seedlings for 7 days, followed by 24 h light exposure for 5 days to examine the rhythmicity of expression for selected circadian clock-related genes. (**B**) The circadian clock was dissected with time-series transcriptomes by detecting the expression rhythmicity of the soybean homologs of circadian clock-related genes: *CCA1*, *ELF3*, *ELF3L*, *ELF4*, *GI*, *LHY*, *LUX*, *PRR3*, *PRR5*, *PRR7*, *RVE1*, *RVE7* and *TOC1*. *CCA1* indicates *GmCCA1* family, *ELF3* indicates *GmELF3* family, etc. The expression levels were normalized to show the circadian rhythmicity. (**C**) Quantitative PCR verification of gene expression rhythmicity of soybean homologs of the known evening-phased evening complex components *ELF3* and *ELF4*. (**D**) Quantitative PCR verification of gene expression rhythmicity of soybean homologs of the known afternoon-phased circadian clock component *GIGANTEA* and clock-related light signaling gene *Phytochrome A*. *GmActin* was used as the reference gene for expression normalization. HPE, hour post entrainment. Although this experiment exposed plants to light constantly, the grey bars indicate the 12-hour periods that would represent nighttime in a 12 h day:12 h night photoperiod

Transcriptomes of the unifoliate leaves sampled on the third continuous light day after entraining seedlings of both cultivars for seven short days (12 h light and 12 h night) (Fig. 1 A), were sequenced from a minimum of 53,600,114 reads to a maximum of 95,131,850, with an average of 72,751,819 reads (Table S1). These reads were mapped to the genome with more than 94.6 % reads mapped to the reference genome, more than 92.1 % reads properly paired and mapped, and more than 91.3 % mapped uniquely (Table S1). In all, the mapped reads were distributed along with the gene density (Fig. S1). Thus, transcriptome sequencing was high-quality.

With these transcriptomal data, the soybean circadian clock was analyzed in detail based on the homologs of known circadian clock components. On the whole, these components had sequential expression peaks in a 24-hour rhythmicity (Fig. 1B). *GmCCA1*s (indicating *GmCCA1* family genes), *GmLHY*s and *GmRVE7*s (*REVEILLE 7*) were morning-phased, peaking at dawn then decreasing until early night. *GmPRR3*s, *GmPRR5*s and *GmPRR7*s were afternoon-phased with the highest expression in the afternoon. *GmELF4*s and *GmELF3*s were evening-phased, peaking at dusk and in the evening, respectively. The aforementioned genes exhibited a consistent expression profile with their counterparts in *Arabidopsis* [12].

However, we found that some circadian clock genes featured different expression profiles from their counterparts in *Arabidopsis*. For example, *GmRVE1*s was evening-phased while *Arabidopsis RVE1* (*REVEILLE 1*) is morning-phased [42]. *GmGI*s peaked around dusk while its *Arabidopsis* counterpart peaked in the afternoon. *GmLUX*s and *GmTOC1*s peaked in the early night while their *Arabidopsis* counterparts peaked around dusk [6]. Noticeably, *ELF3* homologs were divided into two clades. One clade of *GmELF3*s (including *GmELF3a*, *GmELF3b*, and *GmELF3*c) was evening-phased as predicted, while the other clade of *GmELF3L*s was morning-phased (including *GmELF3Ld* and *GmELF3Le*). These results support that the soybean circadian clock diverges from the known *Arabidopsis* canonical model [6].

To further evaluate the transcriptome data and the soybean circadian clock model, we performed a quantitative PCR experiment with three biological replicates to assess the expression of the homologs of the known evening-phased clock components *ELF3* and *ELF4*, and the homologs of the known afternoon-phased clock component *GIGANTEA*, as well as the two genes encoding phytochrome A, which is involved in light signaling of the circadian clock. We found that the *ELF3* homologs *J/GmELF3a*, *GmELF3b, GmELF3c* and *GmELF4a* all showed a robust and very similar circadian clock rhythmicity in both cultivars with a peak in the evening and a trough at dawn, although the functional *J/GmELF3a* was expressed at a higher level in ZH24 than was the nonfunctional *j/Gmelf3a* in HX3 (Fig. 1 C). *GmGIa* (also referred as *E2*) and *GmGIb* were evening-phased in both cultivars, similar to the expression pattern of *GmELF4a* (Fig. 1D). These results are consistent with the transcriptome data and show that our transcriptome data are reliable.

Combined with the observation that the light signaling genes *GmPhyA3* (*E3*) and *GmPhyA2* (*E4*) both exhibited similarly robust rhythmicity in the two cultivars (Fig. 1D), all of the assayed genes displayed a comparable oscillation amplitude in ZH24 and HX3, except for the slight differences in *J/GmELF3a* and *GmGIa* (*E2*), indicating that the expression of all genes showed a similarly robust rhythmicity with consistent phasing in the two cultivars (Fig. 1 C and 1D). These results showed that 7 day entrainment of soybean seedlings generates a self-sustained circadian rhythmicity, and the circadian clock is conserved in two geographically, phenotypically and genetically distinct soybean cultivars, even in the genetic background with the *j/Gmelf3a* dysfunctional allele.

### Soybean genes oscillate in a differentially-phased rhythmicity

**Fig. 2:**
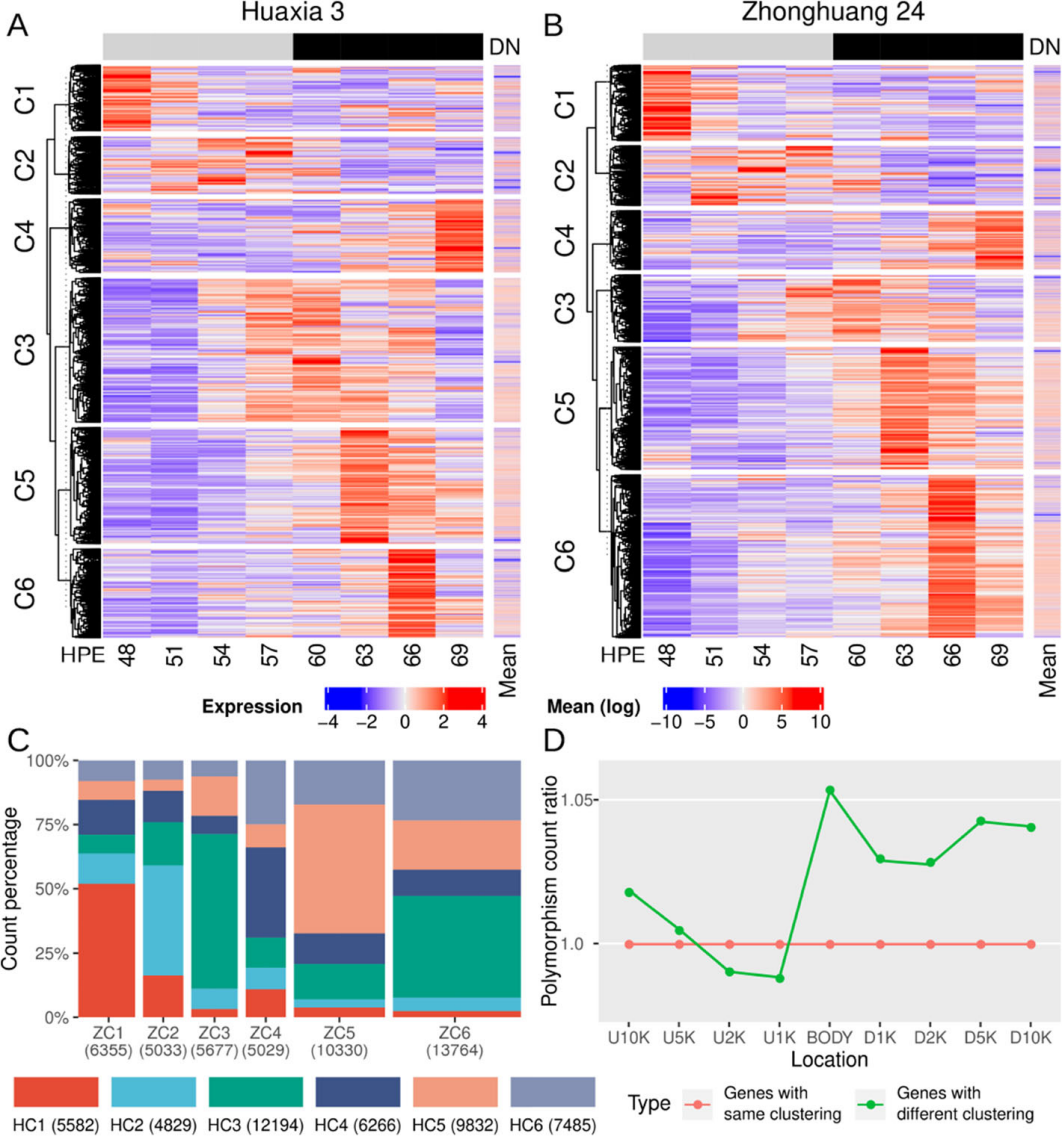
Gene clustering of Huaxia 3 and Zhonghuang 24. (**A** and **B**) Genes are clustered based on scaled expression in Huaxia 3 and Zhonghuang 24. Expression, scaled gene expression. Mean, mean of gene expression (log values); DN, day and night. The black part of the bar indicates the supposed night in continuous light condition. (**C**) Intersection of gene clusters between Huaxia 3 and Zhonghuang 24. Width is scaled to cluster size of Zhonghuang 24. (**D**) Polymorphism count ratio of gene body and its flanking regions. Red shows genes that clustered consistently in Huaxia 3 and Zhonghuang 24. Green shows genes that clustered differently in Huaxia 3 and Zhonghuang 24. The polymorphism count of the genes with the same clustering groups were used as the control. U10K, U5K, U2K, and U1K are the upstream regions of 10, 5, 2 and 1 kilobases, respectively. D1K, D2K, D5K and D10K are the downstream regions of 1, 2, 5, and 10 kilobases, respectively. BODY, gene body

Because soybean has a different circadian clock from *Arabidopsis*, it is necessary to explore the expression patterns of all soybean genes, which is a valuable reference for future studies on gene function in soybean. We performed a clustering analysis to obtain an overview of the rhythmicity of all genes in both cultivars. Soybean genes were grouped into six clusters (C1-C6) with different expression profiles (Fig. 2 A and 2B and Table S2), and the clusters had different numbers of genes from each of the two cultivars. Clusters C1-C6 had 5,582, 4,829, 12,194, 6,266, 9,832 and 7,485 genes, respectively, in HX3, and 6,355, 5,033, 5,677, 5,029, 10,330 and 13,764 genes, respectively, in ZH24; the top two clusters were C3 and C5 in HX3, and C6 and C5 in ZH24 (Fig. 2 C).

The clusters had different expression patterns. Cluster C1 was highly expressed at dawn (H48) and C3 around dusk, and were considered day-night transit clusters (Fig. 2 A and 2B). C2 peaked in the day (H51, H54, and H57) and was a day cluster (Fig. 2 A and 2B). Genes in C5, C6, and C4 were highly expressed in the early (H63), middle (H66) and late night (H69) respectively, and were considered night clusters (Fig. 2 A and 2B). In both cultivars, there were many more genes in the night clusters than in the day clusters, indicating that gene expression is preferentially activated at night. This is consistent with the growth of plants in darkness.

GO (gene ontology) enrichment analysis showed that day-night transit clusters C1 and C3 respectively enriched lipid and carbohydrate metabolism related GO terms, and ribosome biogenesis related GO terms. Day cluster C2 had enriched chloroplast and photosynthesis related GO terms. Night clusters C5, C6 and C4 had enriched protein biosynthesis and metabolism related GO terms, protein phosphorylation related GO terms, and transcription regulation related GO terms, respectively (Table S3). These GO results were consistent with the properties of different clusters. Such results provided a reasonable explanation of catabolism peaking at night and anabolism occurring during the day.

Each cluster had different member genes in the two cultivars. Out of the total of 46,188 detected genes, less than half (19,017, 41.2 %) had the same clustering in the two cultivars (Fig. 2 C). To further elucidate possible reasons for the clustering difference, the distribution of polymorphisms was analyzed. Comparing the genes with the same clustering in the two cultivars, the genes with different clustering exhibited higher polymorphism levels in the upstream 5 K (kilobase) region, the gene body and the downstream regions, but not in the upstream 2 to 1 K regions (Fig. 2D). Thus, clustering differences in the two cultivars may be attributed to genomic variations.

### Soybean has a circadian rhythmicity of alternative splicing

**Fig. 3:**
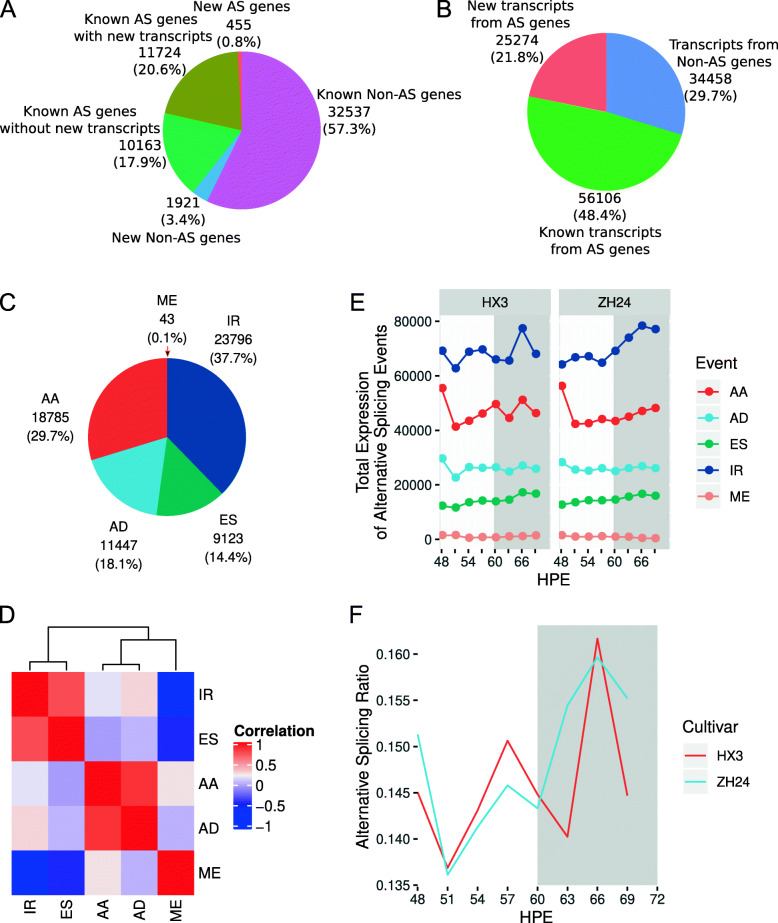
Alternative splicing shows the circadian rhythmicity. (**A**) Statistics of alternative splicing (AS) events in assembled genes. (**B**) Statistics of alternative splicing (AS) events in assembled transcripts. (**C**) Alternative splicing events are counted based on the transcript with the highest expression for each gene. (**D**) Correlations of alternative splicing events. (**E**) Total expression levels of alternative splicing events. (**F**) Ratio of the total expression between alternative splicing transcripts and all transcripts. AA, alternative 3’ acceptor site; AD, alternative 5’ donor site; ES, exon skipping; ME, mutual exon and IR, intron retention. HX3, Huaxia 3. ZH24, Zhonghuang 24. Grey bar, the supposed night in continuous light conditions

As shown before, gene expression exhibited a circadian rhythmicity in soybean, so we wanted to determine whether alternative splicing exhibits some rhythmicity as well. In our time-series RNA-Seq analysis, 56,800 genes were assembled. A total of 34,458 (60.7 %) genes including 32,537 (57.3 %) known genes and 1,921 (3.4 %) new genes were not found to be alternatively spliced. A total of 22,342 (39.3 %) genes had alternative splicing transcripts. Of these, 10,163 (17.9 %) known alternatively spliced genes had no new alternatively spliced transcripts, while 11,724 (20.6 %) known alternatively spliced genes and 455 (0.8 %) new genes had new alternatively spliced transcripts (Fig. 3A). There were 25,274 new alternative splicing transcripts, which accounted for 21.8 % of 115,838 assembled transcripts (Fig. 3B). In all alternative splicing transcripts, there were 23,796 intron retention (IR, 37.7 %), 18,785 alternative 3’ acceptor site (AA, 29.7 %), 11,447 alternative 5’ donor site (AD, 18.1 %), 9,123 exon skipping (ES, 14.4 %) and 43 mutual exon (ME, 0.1 %) events (Fig. 3 C). Taking into account the occurrence of alternative splicing events on an alternative splicing transcript, these five events were grouped into three clades, one included AA and AD, one included ES and IR, and one included ME (Fig. 3D). Moreover, when measured by the total expression of alternative splicing events on alternative splicing transcripts, these events showed a circadian rhythmicity to some extent (Fig. 3E). They appeared to have low expression during the day and high expression at night (Fig. 3E). Around dusk or dawn, the alternative splicing ratio experienced a sharp drop (Fig. 3 F). Moreover, the alternative splicing ratios at night were higher than during the day (Fig. 3 F). This indicated that night is the main time for regulation of gene expression level in soybean.

### Soybean chromosomes are activated in a circadian rhythmicity

**Fig. 4:**
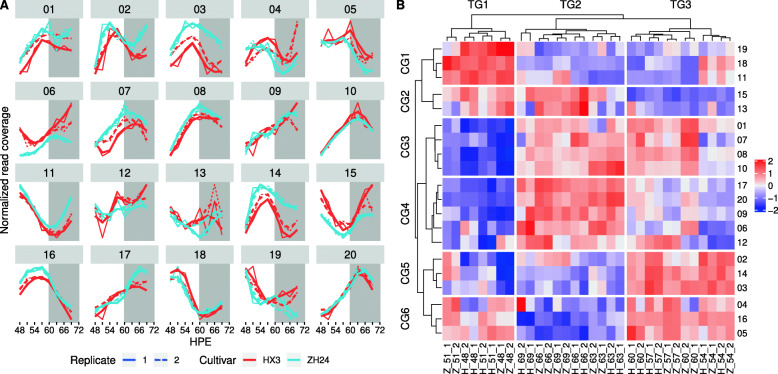
Chromosomes are activated in a circadian rhythmicity. (**A**) Clustering of chromosomes at various time points. (**B**) Normalized read coverage of chromosomes over different time points. Grey bar, the supposed night in continuous light conditions

Thousands of genes are organized in each chromosome. It is thus interesting to elucidate whether a chromosome is activated in circadian rhythmicity. The activation level of a chromosome can be measured as the number of reads mapped on the chromosome. We found that chromosomes exhibited a diurnal rhythmicity (Fig. 4 A). Consistent with the transcriptome sequencing being well replicated, the rhythmicity was robustly similar among replicates. Although the two cultivars are genetically distinct, the rhythmicity of all chromosomes was similar and mostly conserved in the two cultivars. However, three chromosomes – Chr01, Chr03, and Chr07 – differed in the activation level but had similar rhythmicity; Chr06 differed in oscillation phase and amplitude (Fig. 4 A). Moreover, the chromosomes were clustered into six groups (chromosome group, CG) with different expression profiles. CG1 was morning-phased (including Chr11, Chr18 and Chr19); CG2 was dawn-phased (including Chr13 and Chr15); CG3 was evening-phased (including Chr01, Chr07, Chr08 and Chr10); CG4 was night-phased (including Chr06, Chr09, Chr12, Chr17 and Chr20); CG5 was noon-phased (including Chr02, Chr03 and Chr14); and CG6 was afternoon-phased (including Chr04, Chr05, and Chr16) (Fig. 4B). Time points were also clustered into three groups (time-point group, TG). TG1, which included 48 and 51 HPE, is in the morning. TG2, including 63, 66 and 69 HPE, is in the night. TG3, which included 54, 57 and 60 HPE, is in the afternoon (Fig. 4B).

### No genes were consistently differentially expressed over different timepoints

**Fig. 5:**
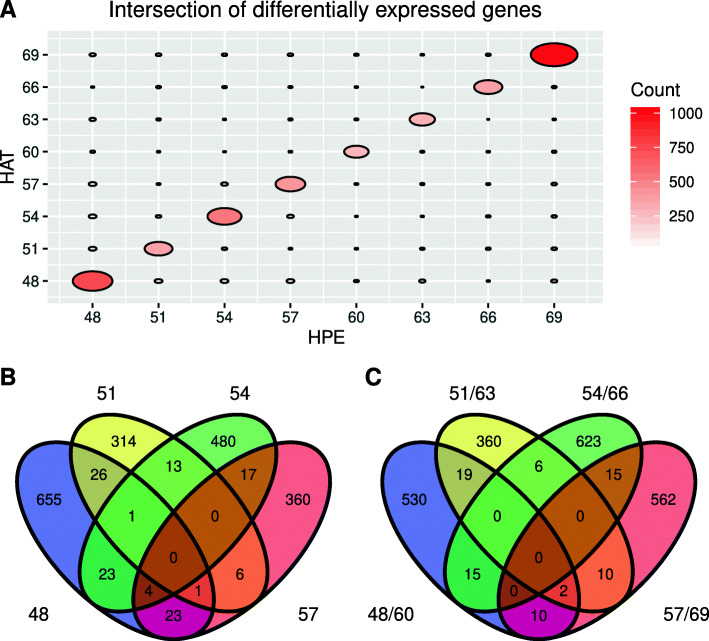
Differentially expressed genes (DEGs) between Huaxia 3 and Zhonghuang 24 change significantly over time points. (**A**) The intersection of DEGs at different time points. The area of an ellipse indicates the number of DEGs that overlapped in two related time points. (**B**) The Venn diagram of DEGs at four time-points, 48, 51, 54 and 57. (**C**) The Venn diagram of DEGs at four time-point combinations, 48/60, 51/63, 54/66 and 57/69. 48/60 means the supposed mixed samples sampled at 48 and 60 h after continuous light treatment. 51/63, 54/66 and 57/69 are similar to 48/60

Because genes exhibited a circadian rhythmicity in the two cultivars, we wanted to figure out the differentially expressed genes (DEGs) potentially responsible for the phenotypic juvenility difference. However, although there were hundreds of DEGs at different time-points, few DEGs overlapped at more than two time-points (Fig. 5 A and 5B). Considering that a multiple-timepoint sampling strategy may be adopted to reduce the fluctuation of peaks and troughs, we simulated a 12-hour spaced sampling strategy and combined two 12-hour spaced samples (for example, 48 and 60 HPE samples) into one merged sample (48/60 HPE sample). We also found that DEGs of merged samples hardly overlapped (Fig. 5 C). These results indicated that DEGs are highly impacted by sampling timing and no conventional DEGs can be reliably claimed to be genes resulting from the phenotypic differences. Therefore, to more thoroughly dissect one gene or one treatment, it is necessary to perform a circadian rhythmicity analysis.

### *GmELF3a* phase- and amplitude-modulates the expression of *GmFT* family genes

**Fig. 6:**
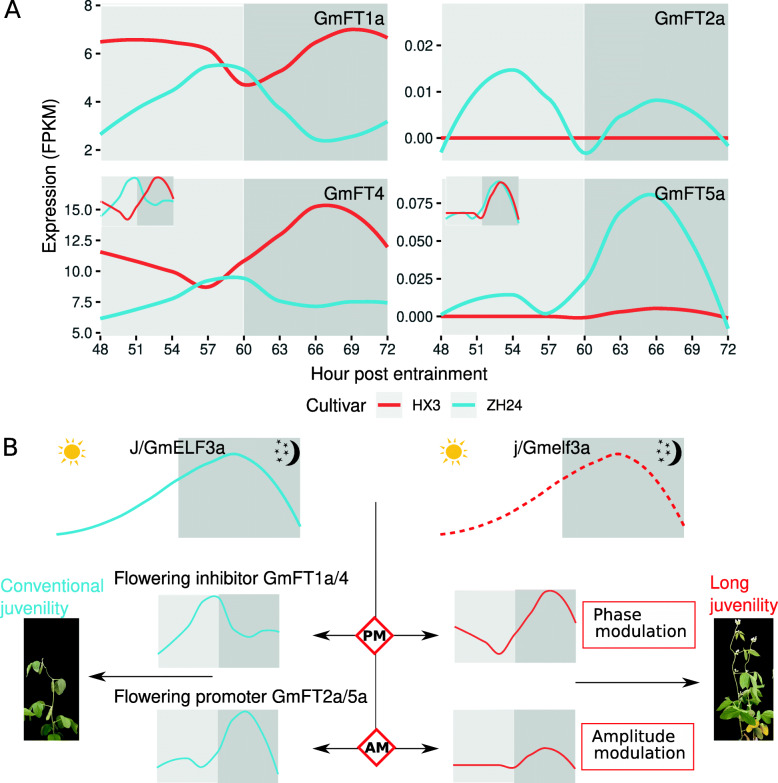
*J/GmELF3a* regulates juvenility and maturity through phase/amplitude modulating of GmFT family genes. (**A**) The expression rhythmicity of the *GmFT* family in two cultivars Huaxia 3 and Zhonghuang 24. The inset shows the rhythmicity phasing of *GmFT4* and *GmFT5a* with the expression levels normalized. (**B**) A model of *J/GmELF3a* regulating juvenility and maturity. The dashed line indicates the unchanged rhythmicity of *GmELF3* homologs in a loss-of-function *GmELF3a* mutant. PM, phase modulation; AM, amplitude modulation

Oscillations have a phase and an amplitude. We further analyzed the oscillation of the flowering-related *GmFT* family, which is homologous to florigen-encoding gene *FLOWERING LOCUS T*. In soybean, the *GmFT* family has essential but divergent functions in flowering regulation, with some members (*GmFT1a*/*4*) inhibiting flowering and some members (*GmFT2a*/*5a*) promoting flowering. We found that these two types of *GmFTs* responded differently in the two cultivars (Fig. 6 A). Both types exhibited obviously different amplitudes of expression vibration in the two cultivars (Fig. 6 A). Flowering inhibitors *GmFT1a*/*4* phased differently in the two cultivars: they phased in the evening in HX3 but in the afternoon in ZH24, while flowering promoter *GmFT5a* phased similarly in the two cultivars (Fig. 6 A). These results indicated that *GmELF3* might phase-modulate the flowering-inhibiting members of the *GmFT* family and amplitude-modulate the flowering-promoting members to regulate the juvenility and maturity traits of soybean (Fig. 6B).

## Discussion

The circadian clock in soybean is different from that in *Arabidopsis*. Quantitative real-time PCR analysis showed consistent results in *ELF3*, *ELF4* and *GI*. One member of *PRR3*/*PRR7*, *GmPRR37*/*GmPRR3b*, also exhibited day-phased expression with a peak in the afternoon, which is consistent with our results [29]. qPCR results and RNA-Seq analysis showed that the expression phases of several circadian clock components in soybean were significantly different from the expression phases of their counterparts in *Arabidopsis*. The results confirmed that the soybean circadian clock is different from the known canonical model proposed in *Arabidopsis* [6]. Moreover, Song et al. [43] suggested it is necessary to fill the gap between laboratory and natural conditions in terms of the circadian clock in soybean. The circadian clock needs to be further explored under natural conditions to help understand its relationship to the maturity trait.

The present study demonstrated that the circadian clock in soybean is conserved even in two geographically, phenotypically and genetically distinct cultivars, indicating that the time-keeping mechanism is inherently required to be stable to properly synchronize biological processes with daily changes. ELF3, a highly conserved plant-specific nuclear protein, is an indispensable component of the circadian clock. ELF3 functions as a scaffold to directly bind ELF4 and LUX to form the EC [12, 13]. ELF3 has essential functions in crops [14–19]. Loss-of-function of *elf3* results in arrhythmic circadian output in *Arabidopsis* and barley [8, 44, 45]. However, in soybean, regardless of the functional status of the *J/GmELF3a* gene, the circadian rhythmicity was regular and similar in two distinct cultivars (Fig. 1), suggesting that the circadian clock was self-sustained in the loss-of-function mutant of *J/GmELF3a*. Similarly, in chickpea (*Cicer arietinum*), another Fabaceae species, the *ELF3* premature mutant did not disturb the circadian clock [46]. Possibly due to evolutionary whole-genome duplication events, *J/GmELFa* have four homologs in the soybean genome, *GmELF3b*, *GmELF3c*, *GmELF3Ld* and *GmELF3Le* (Fig. S2). Phylogenetic analysis showed that two paralogs *GmELF3b* and *GmELF3c* were clustered with *J* into one clade. qPCR and transcriptomal data showed that they exhibited a similar circadian rhythmicity in two cultivars, indicating that the two homologs can function redundantly with *J* to compensate the loss-of-function of *j* in HX3 [25, 26]. Moreover, the two homologs in the other clade exhibited a totally different expression profile. This suggests that *GmELF3Ld* and *GmELF3Le* might have evolved new functional diversity in addition to sustaining the circadian clock.

All detectable genes oscillated with differentially-phased rhythmicity, meaning that no genes were constantly expressed. All genes reached peaks of expression at different time points, and were clustered into six groups, C1-C6. The enrichment of chloroplast and photosynthesis related GO terms in day cluster C2 is consistent with the biochemical and physiological requirements of plants. The protein biosynthesis and transcription regulation related GO enrichment in night clusters indicates that transcription is highly activated to turn over the proteins during the night. Consistently, the majority of genes were included in night clusters, indicating that genes are more active at night and transcription is more likely to take place at night. This makes sense evolutionarily because plants should have enough resources during the day to use light energy for photosynthesis, while at night, plants can make use of material and energy accumulated during the day for transcription and translation.

Alternative splicing events exhibited a circadian rhythmicity as with genes or transcripts, indicating that the circadian clock is involved in post-transcriptional regulation of a gene, which is consistent with Yang et al. [47]. In *Medicago truncatula*, the circadian clock component *MtJMJC5* underwent cold-dependent alternative splicing [48]. Possibly consistent with the high activation level of transcription, the alternative splicing ratios reached a peak in the night. Similar to Yang et al. [47], the most dominant alternative splicing event was IR while the second was AA.

Soybean chromosomes are like genes in that they are expressed with a circadian rhythmicity. Chromosome remodeling might be involved in circadian oscillations, as indicated by work in mammals [49–53]. In mouse liver, Xu et al. [52] found that the chromatin structure protein cohesin regulates circadian gene expression through long-range chromosome interactions, indicating that high-order chromosome structure will impact circadian rhythmicity. For time points, three groups were identified in the present study, TG1, TG2, and TG3. Night time points were in one group (TG2), while day time points were separated into two groups, morning and afternoon. These results indicated that morning and afternoon are representative of different environments to a large extent for soybean growth and development. Interestingly, the key components of the circadian clock were grouped into morning-, day-, and evening-phased. This finding is likely related to the light-quality difference in the morning and in the afternoon.

Due to the oscillation of gene expression, we did not find any consistent DEGs at different time-points. Therefore, we cannot identify which genes are affected by the functional status of *J*/*GmELF3a*. However, when we investigated the circadian rhythmicity of the GmFT family in the two cultivars, we found that flowering-promoting members and flowering-inhibiting members responded differentially to the function of *J*. Based on our results, we proposed a model where *GmFLF3a* phase-modulated the flowering-inhibiting members of the *GmFT* family and amplitude-modulated the flowering-promoting members to regulate the juvenility and maturity traits of soybean. However, more research is required to further elucidate the details.

Considering that nucleotide sequence determines gene expression, the patterning based on dynamic expression data can mimic the evolution based on protein/nucleotide data for homologs of a gene. *GmELF3L*s exhibited a different expression profile from *GmELF3*s, indicating that *GmELF3L*s should have divergent functions from *GmELF3*. There is a similar case in pea (*Pisum sativum*) where two *EARLY FLOWERING3* (*ELF3*) homologs PHOTOPERIOD (PPD) and HIGH RESPONSE (HR) have divergent functions [54]. Thus, our RNA-seq data is a valuable resource to evaluate the functional divergence between homologs.

## Conclusions

The soybean circadian clock is significantly different from the canonical model in *Arabidopsis*, and is conserved regardless of the functional status of *J*/*GmELF3a*. Circadian rhythmicity is exhibited not only at an individual gene level but also at a chromosome level. *GmFLF3a* might phase-modulate and amplitude-modulate the *GmFT* family to regulate the juvenility and maturity traits of soybean. These results and the resultant RNA-seq data should be helpful to understand the soybean circadian clock and elucidate the connection between the circadian clock and soybean maturity.

## Methods

### Plant materials

Two geographically, phenotypically and genetically distinct soybean cultivars, conventional juvenile Zhonghuang 24 (ZH24) and long juvenile Huaxia 3 (HX3) were used. ZH24 is a conventional juvenile soybean cultivar from North China, and HX3 is a long juvenile cultivar from South China. In HX3, a frame-shift mutation of *J* (*GmELF3*) confers the long juvenility trait [26]. These cultivars can be publicly acquired from Chinese Crop Germplasm Resources Information System, Institute of Crop Sciences, Chinese Academy of Agricultural Sciences. Seeds were germinated in soil and seedlings were grown in a growth chamber at 25℃ and a daytime period of 12 h. The light intensity was set as 20,000-lux and the relative humidity was set as 50 %. Seven days after emergence, plants had unifoliate leaves fully expanded and were further treated in continuous light (24 h light/day). Unifoliate leaves (the first true leaves) were sampled and pooled as a biological replicate from three plants every 3 h for 5 consecutive days, and three biological replicates were used at each timepoint. The samples were frozen quickly in liquid nitrogen then stored at -80℃ .

### RNA extraction, cDNA synthesis and real-time quantitative PCR analysis

Total RNA was extracted from the pooled sample leaves following the manual of the TRNzol Universal RNA Extraction Kit (Tiangen, China). cDNA was synthesized with TransScript® One-Step gDNA Removal and cDNA Synthesis SuperMix (Transgen, China). Using the primers listed in Table S4, gene expression was evaluated with real-time quantitative PCR in the Applied Biosystems® QuantStudio™ 7 Flex Real-Time PCR System (Applied Biosystems, USA).

### RNA sequencing, read alignment and expression evaluation

The leaf samples in the third consecutive day were put on dry ice and delivered to Annoroad Gene Technology (Beijing, China). RNA-Seq library preparation and sequencing were performed using the Illumina Hiseq X Ten sequencing platform. The RNA-seq data were deposited into the NCBI SRA database under accession number PRJNA635449. The Williams 82 reference genome and Phytozome annotations (http://www.phytozome.jgi.doe.gov/) were used as the reference for bioinformatics analysis. Clean reads were aligned to the reference genome by HiSat2 with default parameters [55], the transcripts were assembled, and the expression levels were evaluated by Stringtie [55]. The transcriptome related information was extracted and visualized by Ballgown [55]. Alignment statistics were analyzed with SAMtools [56]. Saturation analysis was performed using an in-house script.

### Alternative splicing

After merging all transcript isoforms into one composite gene model, alternative splicing sites were analyzed and counted. The alternative splicing events that occurred at the beginning or end of transcript isoforms were omitted. Consecutive events of exon skipping and mutual exon were counted as one event. Five types of alternative splicing were taken into account, that is, alternative 3’ acceptor site (AA), alternative 5’ donor site (AD), exon skipping (ES), mutual exon (ME) and intron retention (IR). Event expression was measured by the expression level multiplied by the event count of one alternative isoform. The alternative splicing ratio was defined as the ratio between total event expression and total gene expression.

### GO enrichment analysis, KEGG pathway analysis and clustering analysis

Using GO term annotation in AgriGO (http://systemsbiology.cau.edu.cn/agriGOv2/) [57], GO enrichment analysis was performed with the R package TopGO [58]. KEGG pathway analysis was performed with the R package clusterProfiler [59]. Clustering analysis was mainly performed with the R package ComplexHeatmap [60].

## Supplementary Information

**Additional file 1.**

**Additional file 2.**

**Additional file 3.**

## Data Availability

The RNA-seq data is available in the NCBI SRA database under accession number PRJNA635449.

## Abbreviations

AA: Alternative 3’ acceptor site
AD: Alternative 5’ donor site
CCA1: CIRCADIAN CLOCK ASSOCIATED 1
DEG: Differentially expressed gene
ELF3: EARLY FLOWERING 3
ELF4: EARLY FLOWERING 4
ES: Exon skipping
GI: GIGANTEA
GO: Gene ontology
HX3: Huaxia 3 (long juvenility)
IR: Intron retention
LHY: LATE ELONGATED HYPOCOTYL
LUX: LUX ARRHYTHMO
ME: Mutual exon
PHYA: PHYTOCHROME A
PRR5: PSEUDO RESPONSE REGULATOR 5
PRR7: PSEUDO RESPONSE REGULATOR 7
PRR9: PSEUDO RESPONSE REGULATOR 9
RVE1: REVEILLE 1
RVE7: REVEILLE 7
TOC1/PRR1: TIMING OF CAB EXPRESSION 1
ZH24: Zhonghuang 24 (conventional juvenility)
