# Supplementary material for: Transcriptomal dissection of soybean circadian rhythmicity in two geographically, phenotypically and genetically distinct cultivars
Source: BMC Genomics. 2021 Jul 10;22:529. doi: 10.1186/s12864-021-07869-8 (PMC8272290; doi:10.1186/s12864-021-07869-8)
Supplement: Supplementary file 1 — Additional file 1. [file 12864_2021_7869_MOESM1_ESM.pdf]

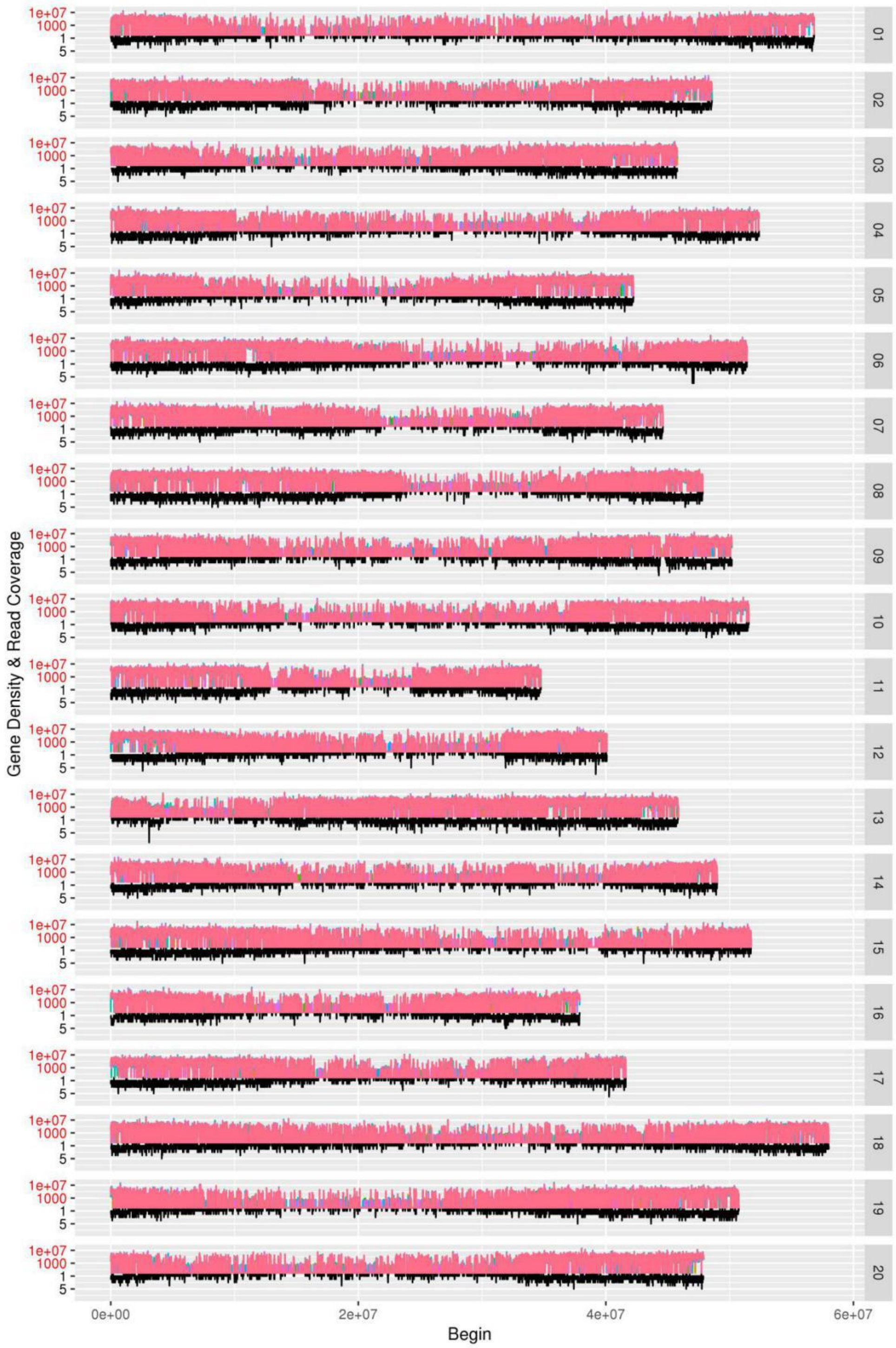

Figure S1. Read coverage and gene density on soybean chromosomes. The upper layer is the distribution of reads on different chromosomes. The lower is the distribution of gene density (gene count/10k bp) on different chromosomes.

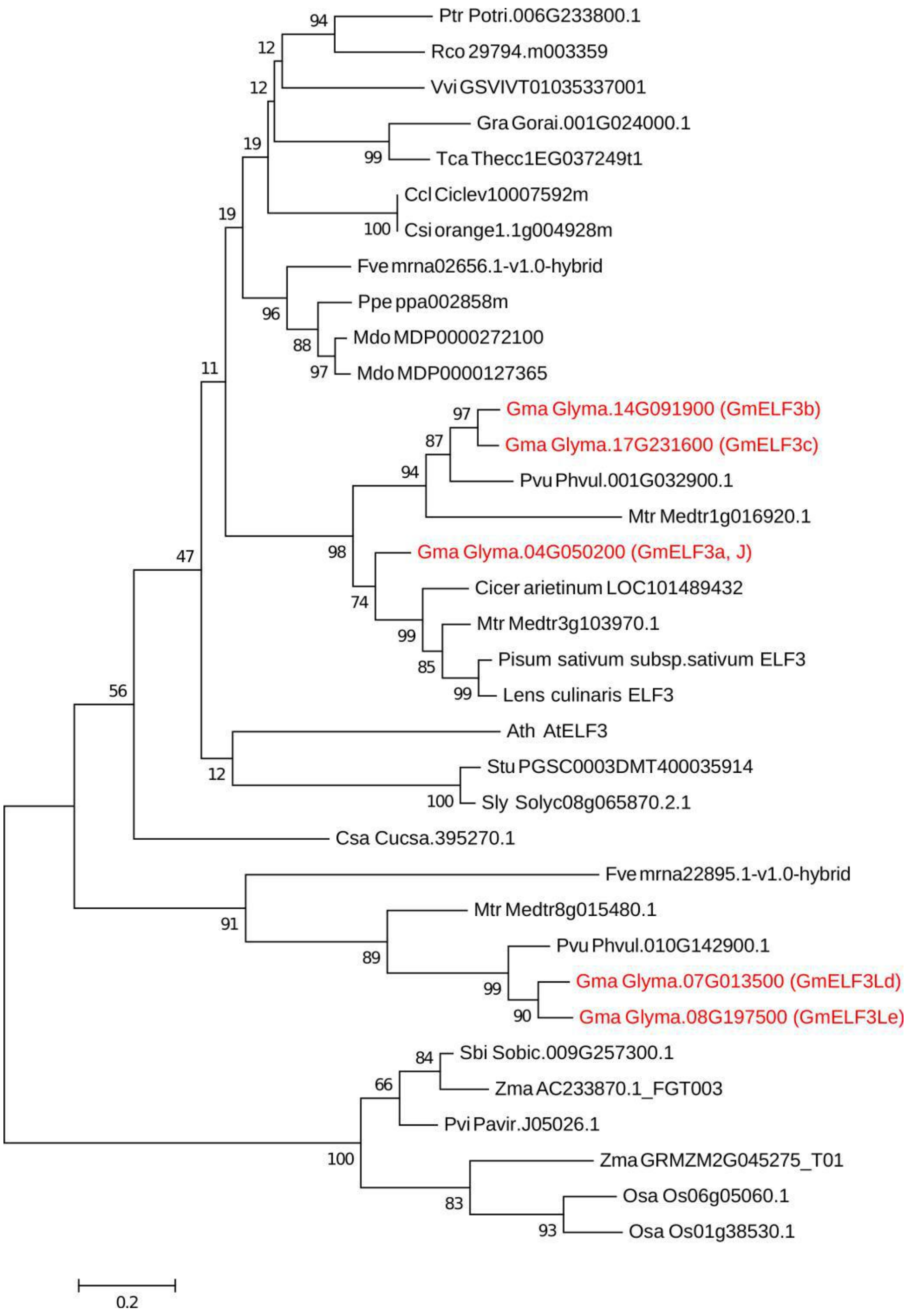

Figure S2. Phylogenetic tree of soybean ELF3 homologs.

Table S1. RNA-Seq statistics of circadian transcriptomes of two soybean cultivars Huaxia 3 (H) and Zhonghuang 24 (Z).

Samples	Total reads	Reads mapped	Reads properly paired	Reads mapped uniquely	Error rate
**H48.1**	82167364	79445245 (96.7%)	77780086 (94.7%)	76635958 (93.3%)	0.0021
**H48.2**	86233344	82560424 (95.7%)	80359614 (93.2%)	79591904 (92.3%)	0.0022
**H51.1**	75982792	73191947 (96.3%)	71564628 (94.2%)	70443614 (92.7%)	0.0022
**H51.2**	85880566	82230140 (95.7%)	80063290 (93.2%)	79351887 (92.4%)	0.0023
**H54.1**	53858244	51715916 (96.0%)	50382696 (93.5%)	50249085 (93.3%)	0.0023
**H54.2**	70864758	67744613 (95.6%)	65919134 (93.0%)	65725045 (92.7%)	0.0023
**H57.1**	53857508	51425989 (95.5%)	50073908 (93.0%)	50000066 (92.8%)	0.0023
**H57.2**	76137880	72852494 (95.7%)	70959694 (93.2%)	70772436 (93.0%)	0.0022
**H60.1**	75452896	71403420 (94.6%)	69558398 (92.2%)	69026105 (91.5%)	0.0023
**H60.2**	54908950	52215532 (95.1%)	50781044 (92.5%)	50725930 (92.4%)	0.0023
**H63.1**	53657344	51516970 (96.0%)	50142266 (93.4%)	49962147 (93.1%)	0.0022
**H63.2**	95131850	91027171 (95.7%)	88546522 (93.1%)	88302151 (92.8%)	0.0023
**H66.1**	54450928	51956881 (95.4%)	50439068 (92.6%)	50273101 (92.3%)	0.0024
**H66.2**	88051260	83574840 (94.9%)	81117566 (92.1%)	80389050 (91.3%)	0.0024
**H69.1**	53987286	51721060 (95.8%)	50270458 (93.1%)	50041475 (92.7%)	0.0023
**H69.2**	83762108	80218509 (95.8%)	78057216 (93.2%)	77502327 (92.5%)	0.0023
**Z48.1**	85978666	83022457 (96.6%)	81036018 (94.3%)	80045090 (93.1%)	0.0020
**Z48.2**	53600114	51608207 (96.3%)	50265464 (93.8%)	49531844 (92.4%)	0.0020
**Z51.1**	64536158	61750522 (95.7%)	60051400 (93.1%)	60107132 (93.1%)	0.0024
**Z51.2**	83553522	80324702 (96.1%)	78210572 (93.6%)	77811520 (93.1%)	0.0020
**Z54.1**	87148214	83475459 (95.8%)	81353244 (93.4%)	80837143 (92.8%)	0.0020
**Z54.2**	72152502	69591678 (96.5%)	68030868 (94.3%)	67464541 (93.5%)	0.0019
**Z57.1**	78487712	75820803 (96.6%)	74085774 (94.4%)	73596818 (93.8%)	0.0019
**Z57.2**	60312444	57846020 (95.9%)	56387028 (93.5%)	56048128 (92.9%)	0.0019
**Z60.1**	87244586	84449048 (96.8%)	82562272 (94.6%)	81897909 (93.9%)	0.0018
**Z60.2**	86550008	83002675 (95.9%)	80875138 (93.4%)	80636660 (93.2%)	0.0020
**Z63.1**	54581774	52623166 (96.4%)	51229328 (93.9%)	51042625 (93.5%)	0.0020
**Z63.2**	83413970	80055764 (96.0%)	77965242 (93.5%)	77724268 (93.2%)	0.0020
**Z66.1**	78785524	75450761 (95.8%)	73400932 (93.2%)	73465109 (93.2%)	0.0020
**Z66.2**	53773000	51710634 (96.2%)	50430826 (93.8%)	50353861 (93.6%)	0.0019
**Z69.1**	79953696	76848923 (96.1%)	74947304 (93.7%)	74694933 (93.4%)	0.0020
**Z69.2**	73601240	70815614 (96.2%)	69168580 (94.0%)	68662435 (93.3%)	0.0018
**Min**	**53600114**	**51425989**	**50073908**	**49531844**	**0.0018**
**Max**	**95131850**	**91027171**	**88546522**	**88302151**	**0.0024**
**Mean**	**72751819**	**69787424.5**	**68000486.81**	**67591009.28**	**0.0021**

Table S4. Primers for RT-PCR

Primer	Sequence
GmACTIN-F	CGGTGGTTCTATCTTGGCATC
GmACTIN-R	GTCTTTCGCTTCAATAACCCTA
GmELF3a-F	CAGGATGGGCAGTCCGATAC
GmELF3a-R	AACACTCTCTGTTGATTGGCA
GmELF3Le-F	ATCCTTGTTTCCACTCCCACTC
GmELF3Le-R	TGAAATCATCTTGCGTGAATTTGGT
GmELF3b-F	CACGATACTCGGACTGGAGG
GmELF3b-R	AAACACACTCTGTTGATTGGCA
GmELF3c-F	TGGAGGCCCAATACAGAAGG
GmELF3c-R	CAAACACACTCTGTTGATTGGCA
GmELF4a-F	GTCGAGTTCGAAGCTCACAAAG
GmELF4a-R	GGGAAGGACGGTCCACTTG
GmGIa-F	GCAGTTGTACTTCAGGCGGA
GmGIa-R	CATCTGTGGCTCGCAGTAGT
GmGIb-F	AGCCACAGATGGAATGCTGG
GmGIb-R	GGCGACACTTTAACAGGTTTGA
GmPhyA3-F	TTGAAACAGCAACGGTGCCA
GmPhyA3-R	TCTCTTCCTCACCCTGCAATG
GmPhyA2-F	AGAGCTCAGCATAACACATGG
GmPhyA2-R	TGCCTGCTTCCCTTAGATAGC
